# Treatment of pigs with endectocides as a complementary tool for combating malaria transmission by *Anopheles farauti* (*s.s.*) in Papua New Guinea

**DOI:** 10.1186/s13071-019-3392-0

**Published:** 2019-03-19

**Authors:** Cielo J. Pasay, Laith Yakob, Hannah R. Meredith, Romal Stewart, Paul C. Mills, Milou H. Dekkers, Oselyne Ong, Stacey Llewellyn, R. Leon E. Hugo, James S. McCarthy, Gregor J. Devine

**Affiliations:** 1 Clinical Tropical Medicine, QIMR Berghofer Medical Research Institute, Herston, QLD Australia; 2 Department of Disease Control, School of Hygiene and Tropical Medicine, London, London, UK; 3 School of Veterinary Science, University of Queensland, Gatton, QLD Australia; 4 Queensland Animal Science Precinct, University of Queensland, Gatton, QLD Australia; 5 Mosquito Control Laboratory, QIMR Berghofer Medical Research Institute, Herston, QLD Australia

**Keywords:** Endectocides, Ivermectin, Malaria control, *Anopheles farauti*

## Abstract

**Background:**

Outdoor, early-biting, zoophagic behaviours by *Anopheles farauti* (*s.s*.) can compromise the effectiveness of bed nets for malaria control. In the Western Pacific region, pigs and dogs represent significant alternative blood sources for mosquitoes. Treating these animals with endectocides may impact mosquito survival and complement control measures. This hypothesis was explored using membrane feeding assays (MFAs), direct feeds on treated pigs, pharmacokinetic analyses and a transmission model.

**Results:**

Ivermectin was 375-fold more mosquitocidal than moxidectin (24 h LC_50 _= 17.8 ng/ml *vs* 6.7 µg/ml) in MFAs, and reduced mosquito fecundity by > 50% at ≥ 5 ng/ml. Treatment of pigs with subcutaneous doses of 0.6 mg/kg ivermectin caused 100% mosquito mortality 8 days after administration. Lethal effects persisted for up to 15 days after administration (75% death within 10 days).

**Conclusion:**

The application of these empirical data to a unique malaria transmission model that used a three-host system (humans, pigs and dogs) predicts that the application of ivermectin will cause a significant reduction in the entomological inoculation rate (EIR = 100 to 0.35). However, this is contingent on local malaria vectors sourcing a significant proportion of their blood meals from pigs. This provides significant insights on the benefits of deploying endectocides alongside long-lasting insecticide-treated nets (LLINs) to address residual malaria transmission.

**Electronic supplementary material:**

The online version of this article (10.1186/s13071-019-3392-0) contains supplementary material, which is available to authorized users.

## Background

The successful deployment of long-lasting insecticide-treated nets (LLINs), indoor residual spraying (IRS), and effective anti-malarials has been responsible for highly significant declines in malaria prevalence around the globe. LLINs are crucial components of many malaria control successes [1], but, despite their mass distribution, transmission persists and is even resurging in some areas. In the Western Pacific, Papua New Guinea and the Solomon Islands account for 92% of the region’s malaria. Between 2010 and 2016, these countries respectively reported a > 400% and > 40% increase in cases [2].

One reason for the suboptimal effectiveness of LLINs is that they are only fully protective when humans are indoors and under their nets. In settings where malaria transmission is mediated by partially zoophagic vectors that exhibit early and outdoor biting behaviours, LLINs are less useful. One major vector that exhibits these traits is *Anopheles farauti* (*s.s*.) This species is responsible for much of the malaria transmission in Papua New Guinea, the Solomon Islands and Vanuatu [3, 4]. It commonly feeds throughout the night, both indoors and outdoors, with a pronounced peak in activity in the early evening [5]. The propensity of *An. farauti* (*s.s*.) to feed on non-human hosts is well-documented, and tends to increase in the presence of LLINs which, by protecting humans, serve to “push” mosquitoes to alternative hosts [6–8]. LLINs will continue to be a mainstay of malaria control in most malaria-endemic areas, but there is an urgent need for their deployment to be complemented by other vector control tools that help combat the behavioural resilience described above. One promising approach is to treat hosts with endectocides that kill mosquitoes that feed on those hosts [9–11].

Endectocides of interest include ivermectin and moxidectin, macrocyclic lactones that have a broad spectrum of activity against nematodes and arthropods, such as ticks, scabies mites and head lice [12]. The potential use of ivermectin-treated hosts to target mosquitoes was first recognized during the mass drug administration (MDA) of ivermectin to humans to combat filariasis [13, 14]. This prompted a number of trials designed to examine impacts on *Anopheles gambiae* (*s.l*.) mortality when exposed to ivermectin-treated humans [15–17] and cattle [18–20]. More recently, the mosquitocidal effect of ivermectin against *An. farauti* was observed during an MDA strategy to control human scabies in the Solomon Islands [21].

Moxidectin is a highly lipophilic, second-generation macrocyclic lactone under development as an alternative to ivermectin for the treatment of onchocerciasis and human scabies [22, 23]. Although others have reported that moxidectin is far less toxic than ivermectin when presented to *An. gambiae* and *An. arabiensis* mosquitoes *via* membrane feeding assays or treated cattle [18, 24, 25], it is more potent than ivermectin against skin burrowing mites in pigs because of its accumulation in subcutaneous tissues [26]. As blood-feeding mosquitoes probe dermal blood vessels or extravasated blood just beneath the skin [27] it is possible that mosquitoes may be unusually exposed when feeding on live pigs.

The operational feasibility of mosquitocidal zooprophylaxis is driven by the intrinsic host-preference of mosquitoes. Previous studies from Papua New Guinea (PNG) have reported that humans, pigs and dogs account for almost all blood meals taken by *An. farauti* (*s.s*.), and that the proportion of each blood resource taken depends on its relative abundance and availability [6, 8, 28, 29]. High LLIN coverage, making human hosts unavailable, appears to push vectors towards other hosts [6, 29]. Dogs are sometimes the preferred host of *An. farauti* and on occasion have been recorded to comprise > 40% of blood meals in PNG, despite their low abundance relative to human and pig populations [29]. For these reasons, we focused our empirical research and modelling on a hypothetical malaria transmission system mediated by *An. farauti* (*s.s*.) utilising three main blood resources: humans, pigs and dogs.

Predicting the efficacy of an endectocidal zooprophylactic treatment will rely on identifying the most pragmatic or effective route of drug administration, its pharmacokinetic properties in the target host and its pharmacodynamic effect on the target. Here, we investigated the impact of ivermectin and moxidectin on blood-feeding *An. farauti* (*s.s*.) using a combination of membrane feeding assays and direct feeds on treated pigs. Comparisons were made between drug levels in the blood resulting from subcutaneous (SC) injection, topical treatment, and oral treatment. Using the mortality data derived from the SC treatment, the effect of endectocide coverage and host choice was explored using an adapted transmission model [30, 31]. The model was used to simulate the impact of endectocidal treatments on both *Plasmodium falciparum* and *P. vivax* malaria transmission for range of host preferences and endectocidal coverage. Projections were made for the transmission reductions that might be achieved and the potential to include the ivermectin treatment of domestic animals as part of an integrated vector management plan for malaria control.

## Results

### Membrane feeding assays (MFAs)

**Fig. 1:**
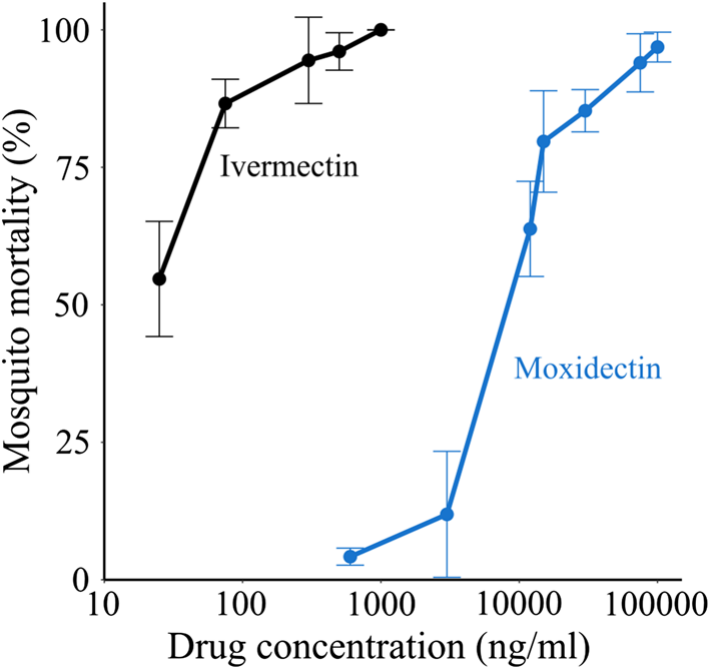
In vitro activity of ivermectin and moxidectin 24 h post-membrane feeding. Lower concentrations of ivermectin than moxidectin are necessary to achieve the same mortality level in mosquitoes

Results of MFAs showed a 375-fold difference in activity: 24 h LC_50_ of 17.8 ng/ml (95% CI: 13.6–22.7 ng/ml) and 6700 ng/ml (95% CI: 5000–8700 ng/ml) for ivermectin and moxidectin respectively (Fig. 1). Below 3000 ng/ml, moxidectin had no impact on mosquitoes.

**Fig. 2:**
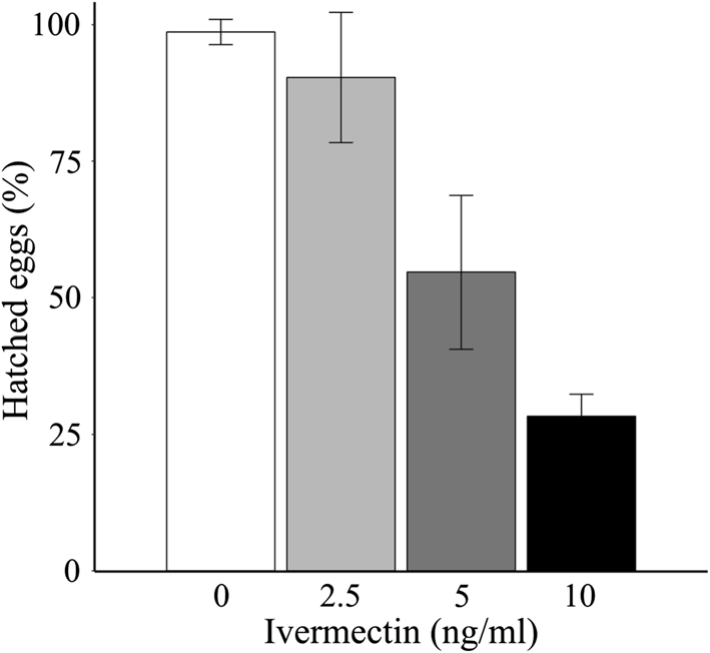
Effect of sublethal ivermectin concentration on mosquito fecundity. The proportion of hatched eggs decreased as the ivermectin concentration increased in the blood meal of mosquitoes

Further MFAs were undertaken to investigate the sublethal effects of ivermectin on mosquito fecundity. Eggs laid by female mosquitoes that had fed on 5 and 10 ng/ml ivermectin exhibited significantly decreased hatching rates (53.2% and 27.0%, respectively, *P* = 0.0053 (Fig. 2). The number of eggs laid was unaffected.

### Pharmacokinetics and impact of live feeds

**Fig. 3:**
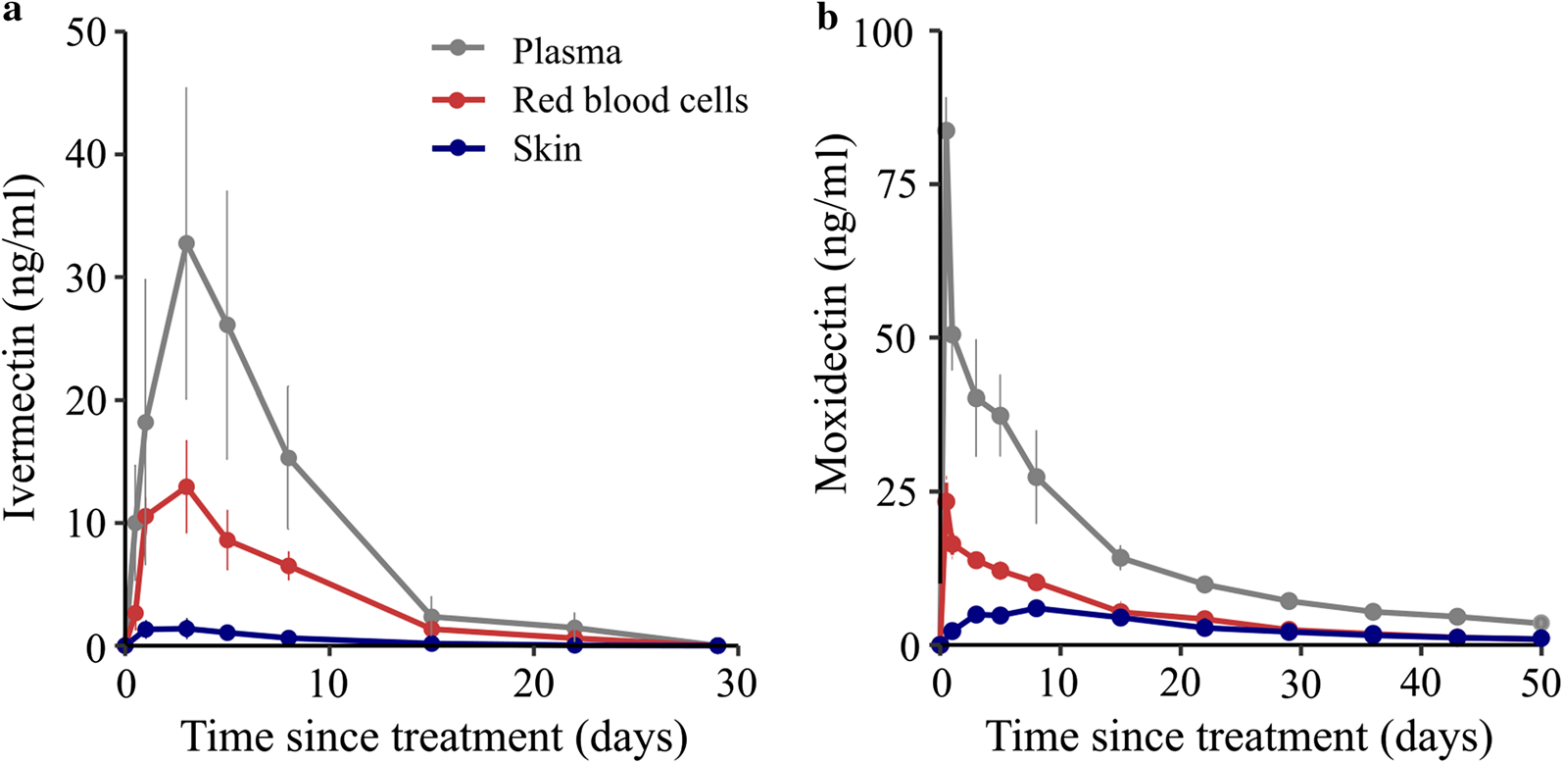
Pharmacokinetic profiles for plasma, red blood cells, and skin. Ivermectin and moxidectin attained the highest concentrations in plasma and lowest in skin (*n* = 2 pigs per condition, dose = 0.6 mg/kg ivermectin or moxidectin)

Pharmacokinetic assays on pigs treated with 0.6 mg/kg of drug by SC injection showed consistently higher levels of ivermectin and moxidectin in the plasma compared with red blood cells or skin biopsies (Fig. 3, Additional file 1: Table S1). The maximum concentration (C_max_) and half-life (t_1/2_) of ivermectin in blood (plasma plus red blood cells) was 34.1 ng/ml and 4.0 days, respectively. The values for moxidectin were 83.7 ng/ml and 19.4 days, respectively (Table 1). Ivermectin levels in treated pigs reflected the concentrations required to cause 24 h mortality observed *in vitro* (MFAs) while moxidectin levels did not.

**Table 1:** Summary of pharmacokinetic data in blood (plasma plus red blood cells) for pigs treated with 0.6 mg/kg of ivermectin or moxidectin

Delivery method	Ivermectin			Moxidectin
	Pour-on^a^	Oral^a^	Subcutaneous^a^	Subcutaneous
AUC_0-t_ (ng/ml∙d)	4.4 (3.4, 5.4)	48.3 (47.7, 52.8)	246.9 (218.3, 275.6)	686.7 (623.9–749.4)
AUC_0-**∞**_ (ng/ml∙d)	5.9 (5.9, 5.9)	48.5 (43.9, 53.0)	256.6 (236.1, 277.0)	786.0 (745.0–827.0)
C_max_ (ng/ml)	0.9 (0.5, 1.2)	19.9 (19.1, 20.7)	34.1 (26.4, 41.7)	83.7 (87.5–79.9)
T_max_ (d)	3.0 (3.0, 3.0)	1.0 (1.0, 1.0)	2.0 (1.0, 3.0)	0.5 (0.5–0.5)
t_1/2_ (d)	6.1 (8.7, 3.4)	1.7 (1.8, 1.7)	4.0 (5.4, 2.6)	19.5 (21.5–17.4)^b^

^a^Data are presented as a “Mean (Pig 1, Pig 2)”

^b^The moxidectin terminal half-life in blood may not be appropriate as the time interval the drug was measured over was not sufficient (< 2t_1/2_). Therefore, caution is required when interpreting the terminal half-life for moxidectin

**Fig. 4:**
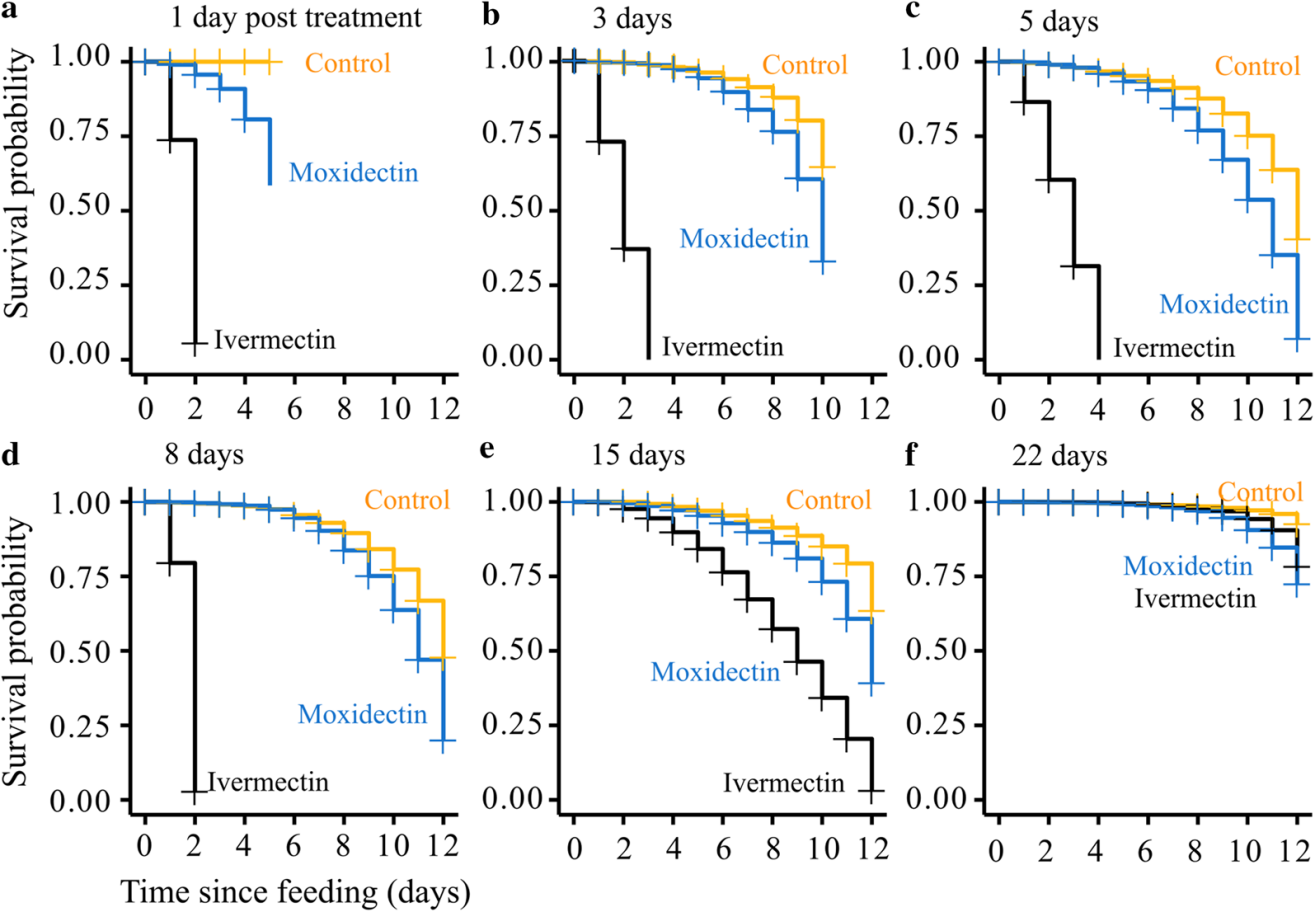
Mosquito survival probability after direct feeding on ivermectin- and moxidectin-treated pigs. **a**–**d** At week one post-treatment of pigs (by SC injection of ivermectin and moxidectin), mosquitoes were allowed to feed on pigs on Day 1 (**a**), Day 3 (**b**), Day 5 (**c**) and Day 8 (**d**) post-treatment. Mean ivermectin plasma concentration detected in pigs on these feeding days was 18.2, 32.8, 26.1 and 15.3 ng/ml, respectively. Mosquito mortality after feeding was recorded and expressed as survival probability. Zero survival probability of mosquitoes was recorded within 2–4 days of feeding on ivermectin-treated pigs. Survival probability of mosquitoes that fed on moxidectin-treated pigs only increased at Day 1 (**a**) and Day 3 (**b**) post-treatment with mean drug plasma concentration of 50.5 and 40.2 ng/ml, respectively. Mosquitoes that fed on moxidectin-treated pigs were frozen and checked after Day 5 (**a**) and Day 10 (**b**) of monitoring to ensure presence of moxidectin (data not shown). From Day 5 and onwards (**c**–**f**), post-treatment of pigs with moxidectin, mean plasma concentrations of 37.4, 27.4, 14.2, 9.8 ng/ml, respectively, had no impact on mosquito survival. **e** At week two or Day 15 post-treatment of pigs, mosquito survival probability of 0 was observed on Day 12 after feeding on ivermectin-treated pigs with decreased mean plasma concentration of 2.4 ng/ml. **f** At week three or 22 days post-treatment of pigs, very low mean ivermectin plasma concentration of 1.45 ng/ml had no impact on mosquito survival. Survival probability was similar to mosquitoes that fed on moxidectin-treated pigs and untreated pigs (control)

*In vivo*, mosquitoes that fed on ivermectin-treated pigs (*via* SC injection) - 1, 3, 5 and 8 days post-treatment, died within 2–4 days. The mean plasma levels for these time points were 18.2, 32.8, 26.1, and 15.3 ng/ml ivermectin, respectively (Fig. 4). Fifteen days after treatment, the mean ivermectin plasma level had fallen below the 24 h LC_50_ (calculated from the MFA data) to just 2.4 ng/ml, but mosquito survival remained significantly lower than controls at all time points 3–12 days after feeding (Fig. 4e). The effect of ivermectin treatment was insignificant 22 days post-treatment of pigs (mean plasma level = 1.4 ng/ml). In comparison, the mean moxidectin plasma levels were always too low to affect mosquito survival.

**Fig. 5:**
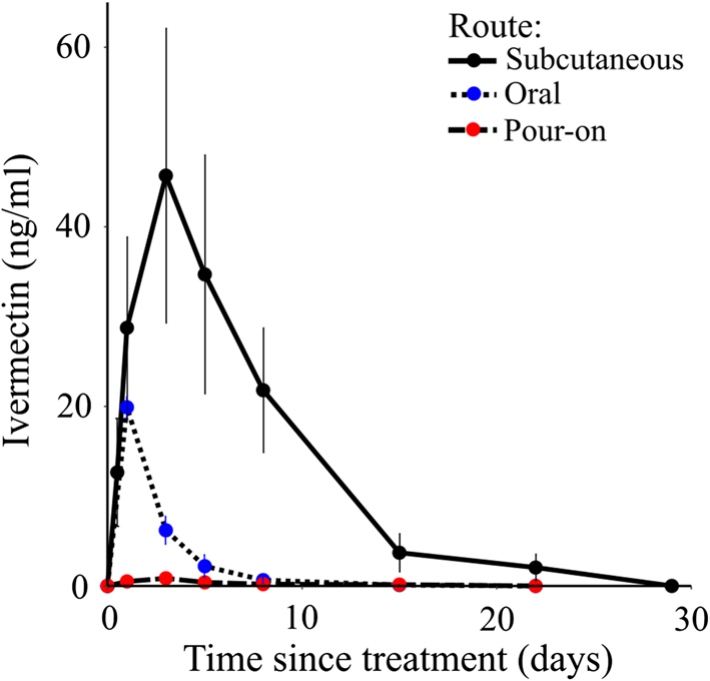
Blood concentrations of ivermectin in pigs delivered *via* subcutaneous injection, oral and pour-on routes. Subcutaneous injection of 0.6 mg/kg ivermectin in experimental pigs resulted in much higher mean blood levels of the drug (18.2, 32.8, 26.1 and 15.3 ng/ml on week 1 and 2.4 ng/ml on week 2, (concentrations found lethal to mosquitoes) and longer residence time (up to 3 weeks) as compared to same drug concentration of 0.6 mg/kg delivered *via* oral and pour-on routes in experimental pigs. The mean lethal dose of 19.9 ng/ml delivered *via* oral route was detectable only after 24 h post-treatment of pigs and rapidly declined to 6.2 ng/ml after 3 days. This lethal dose was not reached by pour-on delivery

The effects of ivermectin delivery were also compared. When 0.6 mg/kg was administered by oral or pour-on routes, blood levels were 66% and 97% lower in comparison to the levels observed after subcutaneous injection to pigs (Fig. 5, Table 1, Additional file 1: Table S2). Delivered orally, the mean blood level of ivermectin in pigs rose to19.9 ng/ml within 24 hours of treatment but declined to 6.2 ng/ml after 3 days and was not detectable after 7 days. Pour-on delivery resulted in even lower blood levels (0.15–0.5 ng/ml).

No mosquito live feeds were performed on pigs treated *via* oral and pour-on routes, instead, we modelled a range of doses to determine the dose necessary to achieve a commensurate impact with SC injection. Results showed that equivalent bioavailability would be achieved at 1.0–3.2 mg/kg *via* the oral route and 24.0–33.7 mg/kg *via* pour-on routes (Additional file 1: Table S3). High variability in dose was observed between the three pharmacokinetic parameters used for the equivalence calculations. As data were available for just two pigs for each of the oral, pour-on, and subcutaneous delivery methods, a larger sample size would be needed to make more confident estimates of the required doses.

### Modelling outcomes

The impact of the strategy was estimated by calculating the relative entomological inoculation rate (EIR) from simulated populations of treated *versus* untreated populations of pigs and dogs. In the absence of any treatment, the annual EIR value was set to a conservative 100 infectious bites per year. This reflects annual EIR estimates of 100–1000 prior to LLIN roll-out in PNG [4].

**Fig. 6:**
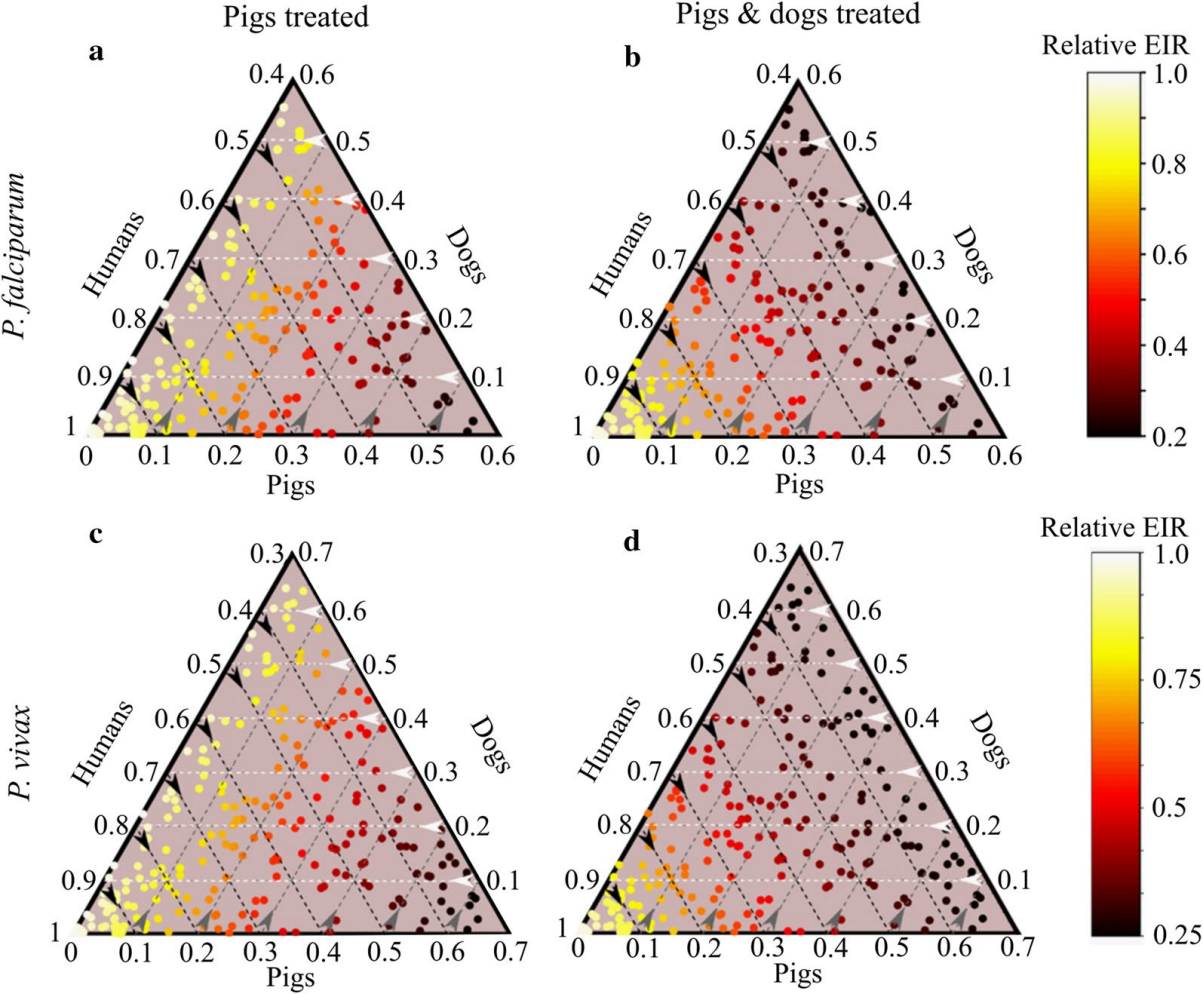
Impacts of endectocidal treatments on entomological inoculation rates (EIR) of *P. falciparum* (**a-b**) and *P. vivax* (**c-d**) following three months of fortnightly endectocidal (ivermectin) applications to pigs alone (**a, c**) or both pigs and dogs (**b, d**). Each point within the triangles refers to a relative EIR value that corresponds to a particular distribution of blood meals (human, pigs or dogs). In the absence of any control, annual EIR was set to equal 100 (Reimer, et al. [4]). Note the minimum proportion of human blood meals was 0.4 and 0.3 for *P. falciparum* and *P. vivax* to produce R_0_ > 1. Within those limits, the relative EIR could be dramatically influenced by host choice. For example, in panel **a**, a mix of blood meal proportions from humans (0.4), treated pigs (0.4) and untreated dogs (0.2) results in a shift in relative EIR to 0.35

The results shown in Fig. 6 are predicted reductions in EIR at the end of the 3-month period of treatment, relative to a no-control scenario. Malaria transmission could not continue (R_0_ < 1) if fewer than 40% and 30% of blood-meals were taken from humans for *P. falciparum* and *P. vivax*, respectively. After 3 months of treatment, the *P. falciparum* and *P. vivax* models predicted significant declines in EIR for a range of host-choice scenarios. Treating pigs alone, and, for example, assuming a human: pig: dog host choice ratio of 0.4: 0.4: 0.2 [6, 8] delivers a dramatic decrease in relative EIR (0.35).

## Discussion

The endectocide ivermectin, when present in mammalian blood and tissues, is widely recognised as having insecticidal activity against blood-feeding anopheline mosquitoes. Its short plasma half-life and operational challenges of mass administration to humans at effective doses [17] suggest that its mosquitocidal feasibility in humans may be limited. Treatment of alternative hosts such as domesticated animals, using higher doses of ivermectin, with better residual activity, may offer more practicable means of implementation [32–34].

One potential target of such a strategy is *An. farauti* (*s.s*.), a partially zoophagic mosquito and a major vector of both *P. falciparum* and *P. vivax* in large parts of Papua New Guinea and the Western Pacific [35]. The first demonstration of ivermectin impacts on *An. farauti* (*s.s*.) was from a treated (0.250 mg/kg) human volunteer. Blood-fed mosquitoes were affected up to 14 days after drug administration [36]. That was the first paper to report on the potential impact of endectocidal zooprophylaxis in pigs. In this paper, we implemented an empirical and modelling study of that vector control strategy using a combination of membrane feeding assays, live feeds on treated pigs, pharmacokinetic analysis and a transmission model.

Single subcutaneous injection of ivermectin in pigs caused serious impacts on *An. farauti* (*s.s*.) within two weeks of treatment. These outcomes would have profound effects on vector populations, biting rates and entomological transmission parameters. Mosquitoes dying within 3 days will not survive the gonotrophic cycle [5] and are therefore reproductive dead ends. Mosquitoes dying within 10–12 days will not complete the sporogonic cycle (extrinsic incubation period or EIP) of the parasite, and therefore will not transmit malaria. A common measure of the intensity of malaria transmission is the entomological inoculation rate (EIR). This estimates the number of infectious bites per person per unit time [37], and is usually calculated from measures of the number of bites per person per day and the fraction of those bites that are infectious (the “sporozoite rate”). Daily survival alone and in relation to EIP will have profound effects on those parameters and the EIR. Our research has focused on *An. farauti* (*s.s*.) which is the major coastal vector throughout PNG [38]. In the presence of LLINs it is likely to be diverted to pigs; a characteristic which it shares with other established malaria vectors such as *An. punctulatus* [6]. Another partially zoophagic species, *An. koliensis*, may be a poorer target for the ivermectin treatment of domestic animals: the limited literature suggests that humans are always this species’ favoured blood resource [6, 8] but that may make them more vulnerable to the impacts of LLINs.

Our results are similar to those reported for ivermectin-treated cattle, and their impacts on *An. gambiae* (*s.l.*), despite the fact that pigs may exhibit lower C_max_ and AUC values for ivermectin as a result of increased sequestration in the fat tissues and more rapid decreases in plasma levels [39]. SC injection of cattle with 0.6 mg/kg affected blood-feeding *An. gambiae* (*s.l*.) for two weeks after treatment (> 90% death within 10 days) [18], while subcutaneous injection with 0.2 mg/kg affected *An. coluzzi* for three weeks (> 75% death within 12 days) [20].

Membrane feeding assays performed in this study also suggest that blood concentrations of 5 ng/ml will reduce egg-hatch rates by > 50% in *An. farauti.* The impacts of ivermectin on the fecundity and fertility of *Anopheles* mosquitoes have been widely reported [18, 40] and may further reduce population density by affecting mosquitoes that survive the gonotrophic cycle and lay eggs. In focusing on the impacts of endectocide-associated mortality on EIR, and not including impacts on fecundity, it is likely that the sizeable reductions in transmission achieved in our model simulations represent conservative estimates.

We had hypothesised that the ivermectin-related macrocyclic lactone moxidectin might be unexpectedly effective against *An. farauti* because of its pronounced lipophilicity, its remarkable efficacy on skin burrowing mites in pigs [26], and the fact that blood-feeding mosquitoes probe dermal blood vessels and extravasated blood just beneath the skin [27]. However, although moxidectin had a more favourable pharmacokinetic profile than ivermectin in pigs, it did not reach high concentrations in the skin, and had no impact on mosquito mortality. We therefore corroborate a report from cattle that shows no impact of moxidectin on *An. gambiae* at 0.6 mg/kg [18].

Subcutaneous administration of ivermectin to animal herds using an injection gun is fast and simple (assuming that animals can be adequately contained) but other routes of drug administration are of interest, particularly for free-ranging or feral animals. We compared the pharmacokinetics of SC, topical and oral administration (0.6 mg/kg) and determined that topical application was the least efficient delivery method while oral treatment would require delivery at 1–3 mg/kg to achieve equivalent levels of control. Although this is higher than the usual therapeutic dose of 0.3 mg/kg, it is well within the tolerable range for pigs (toxicity not observed until the dose exceeds 30 mg/kg [41].

Our model allowed for three hosts (humans, pigs and dogs) because they are the dominant resources that have been identified by the few studies conducted in Papua New Guinea that have conducted an unbiased blood meal survey [6, 8, 29]. Pig production is a traditional small-holder activity in PNG, with 50% of all rural households owning one or more pigs that graze or forage around the home [6, 42, 43]. Dogs are also very common in all villages with approximately one dog for every four humans [6]. The presence of LLINs and the availability of pigs and dogs can combine to reduce mosquito predation on humans and to increase the proportion of blood meals taken from alternative hosts [6, 29]. Pigs are the most important alternative host in terms of the proportion of blood meals taken by *An. farauti* (*s.s*.) but dogs can also contribute significantly (20–30% of blood meals) [6, 8]. Overall pigs and dogs can account for well over 50% of blood meals [6, 29].

Although the empirical data from this paper relate to the treatment of pigs, dogs are another potential resource to be treated. Following oral ivermectin administration (0.6 mg/kg) the C_max_ in dogs was consistently > 300 ng/ml and plasma concentrations of > 5 ng/ml were noted until day 18 after treatment [44]. On that basis, we can assume that oral ivermectin treatment of dogs would be at least as effective as SC treatment of pigs.

The feasibility of treating animals with endectocides at fortnightly intervals in rural settings is untested, but it is possible that the practicability of the idea could be improved by involving members of the target community [45] or the development of longer lasting ivermectin formulations [33]. The application of ivermectin to domestic animals for malaria control also requires appraisal of a number of non-target issues. One attractive outcome of endectocidal zooprophylaxis may be the coincidental improvement of animal health through impacts on helminths, ticks and mites. In Pakistan, treatment of cattle, sheep, and goats with topical pyrethroids reduced malaria incidence but also improved animal yields - thereby encouraging community participation in the programme [46]. A less desirable consequence might be the selection of drug resistance in helminths following continual treatment. Although this is a particular risk in intensive livestock farming, selection pressures may be reduced in populations where there is no importation of resistant parasites, treatment is temporally or spatially limited and anthelminthic classes are rotated to reduce selection pressure. These measures can be so effective that, with careful management, ivermectin efficacy can be restored even in intensively farmed herds [47].

## Conclusions

In this study, we investigated the impact of endectocidal zooprophylaxis on *An. farauti* (*s.s*.) feeding on pigs. *Anopheles farauti* (*s.s*.) is the major vector of malaria across much of Papua New Guinea and has a partially exophilic, exophagic, early-biting and zoophagic behaviour. In this region, pigs and dogs contribute a high proportion of blood meals to that vector species. Our three-host model suggests that ivermectin treatment of those alternative hosts will have a profound effect on EIR and, therefore, on the transmission of both *P. falciparum* and *P. vivax* malaria. The approach will be complementary to LLIN use where those nets do not offer full protection against some vector behaviours and where they serve to “push” host-seeking mosquitoes to alternative hosts.

## Methods

### Study design

This study was conducted in two parts: *in vitro* studies at QIMR Berghofer, Brisbane, Queensland, and *in vivo* studies at the Queensland Animal Science Precinct (QASP), Gatton, Queensland, Australia.

### *Anopheles farauti* mosquitoes

The *An. farauti* (*s.s*.) colony was derived from specimens collected in Rabaul, Papua New Guinea in 1972. Permission to release this mosquito from quarantine was granted by the Australian Department of Agriculture Fisheries and Forestry in 2012. This made it suitable for feeding on treated pigs at the Gatton facility. The mosquito colony was maintained at QIMR Berghofer as previously described [48].

### Preparation of drugs for membrane feeding assays

Ivermectin (Sigma-Aldrich 18898, St. Louis, MO, USA) or moxidectin (Sigma-Aldrich 33746, St. Louis, MO, USA) in powder form, were initially dissolved in DMSO to prepare stock concentrations of 10 mg/ml, and aliquots frozen at -20 °C. On the day of mosquito feeding, a frozen drug aliquot was thawed and diluted in PBS to the required concentrations. Each drug concentration for testing was added to anti coagulated blood (1 part drug: 99 parts blood) collected from a human volunteer to reach a range of concentrations (ivermectin: 25–1000 ng/ml; moxidectin: 600–100,000 ng/ml).

### Phase I: Membrane feeding assays

#### Dose-finding and mosquito mortality

Four- to 7-day-old female *An. farauti* mosquitoes were starved overnight prior to feeds. At least 20 mosquitoes were placed in gauze-sealed feeding cups. Feeding cups were prepared in triplicate per drug concentration and control. One ml of drug-spiked blood was delivered in glass feeders attached to a circulating water bath (37 °C), and mosquitoes were allowed to feed on the blood through bovine caecum membranes for 30 min or until engorged. Afterwards, unfed mosquitoes were removed and blood-fed mosquitoes were maintained on 10% sugar solution in the QIMR Berghofer Insectary for 7 days while daily mortality was recorded.

#### Sublethal concentrations of ivermectin and mosquito fecundity

To investigate the effect of sublethal concentrations of ivermectin on *An. farauti*, human blood was spiked with sublethal concentrations of ivermectin (2.5, 5 and 10 ng/ml) and female mosquitoes were allowed to feed through membranes for 30 min. Afterwards, unfed mosquitoes were removed from the cups and batches of fed mosquitoes were maintained on 10% sugar solution, transferred to cages with oviposition cups provided. Mosquitoes were monitored for 5 days after feeding and then removed from the cages. On day 10 the number of hatched eggs (larva) and total numbers of eggs in the oviposition cups were counted.

### Phase II: Pharmacokinetics and live feeding

#### Treatment

Five 6-week-old pigs weighing 10–11 kg were randomised to ivermectin (*n* = 2) and moxidectin (*n* = 2) treatment groups, with one pig serving as a no treatment control. Treated pigs were injected subcutaneously with either Ivermectin (Ivomec, Merial Ltd, Duluth, Georgia, USA) or Moxidectin (Cydectin, Virbac, Sydney,NSW, Australia) at 0.6 mg/kg body weight.

#### Blood draw and skin biopsy

Each pig was mildly sedated with 0.5–0.8 ml of Azaperone (Stresnil, Elanco, Greenfield, Indiana,USA) and laid on a cradle to facilitate blood sampling and conduct of direct mosquito feeds. At least 1 ml blood was extracted from all pigs at 0 hour (before treatment), 6 hours, and Days 1, 3, 5, 8, 15, 22 and 28 after treatment. After collection, plasma was separated from red blood cells and stored frozen. Skin punch biopsy (5 mm) was also performed (under local anaesthetic) at the same time starting from Day 1 onwards to Day 28. Blood draws and skin biopsies were continued for moxidectin-treated pigs up to Day 50 post-treatment. Blood and skin samples were sent to the Department of Chemical Pathology, Royal Brisbane Hospital, for pharmacokinetic analysis.

#### Pharmacokinetic analysis

Ivermectin and moxidectin levels were determined from plasma, red blood cells and skin biopsy samples collected from experimental pigs treated by subcutaneous injection at each sampling point by reverse phase isocratic ultra-performance liquid chromatography (UPLC) coupled with fluorescence detection (Waters Corporation, Milford, MA, USA) following the manufacturer’s protocol. Briefly, blood samples (plasma and red blood cells) were deproteinised and extracted with 1 ml 100% methanol. The samples were centrifuged after and the supernatant dried under air. Pig skin samples, were frozen and then microtomed to produce slices approximately one cell across. The frozen sections were then suspended in 1 ml of 100% methanol and ultrasonicated for one hour. All analytes were then derivatised with N-methylimidazole and trifluoroacetic anhydride and 1 µl was injected onto the UPLC system. Internal standards: ivermectin (Sigma-Aldrich, 18898, St. Louis, MO, USA) and moxidectin (Sigma-Aldrich 33746, St. Louis, MO, USA) in powder form were diluted with DMSO to prepare the calibration curve. Ivermectin and moxidectin were measured against a four-point calibration curve with drug levels expressed in ng/ml. Inter-run imprecision (% CV) across three levels of Quality Control were <8%.

Ivermectin levels were determined from whole blood of treated pigs *via* oral and pour-on routes when drug delivery methods were investigated.

#### Mosquito feeding

Mosquito feeding was also performed at each sampling point starting from Day 1 post-treatment of pigs *via* SC injection. At least 20 female mosquitoes per cup (in triplicate cups) were starved overnight prior to direct feeding on experimental pigs. Mosquito cups were placed on the pig’s underbelly and allowed to feed for 15 min or until engorged. Mosquitoes were also allowed to feed on control pig that did not receive any treatment. After feeding, unfed mosquitoes were removed from the cups and fed mosquitoes were maintained on 10% sugar solution at QIMR Berghofer Insectary, and mortality monitored daily for 12 days.

### Ivermectin *via* oral and pour-on routes of delivery

To investigate the pharmacokinetic profile following other methods of ivermectin administration to pigs, four experimental pigs were treated with the same dose (0.6 mg/kg) of ivermectin *via* oral route (Ivomec Liquid, Merial Ltd; *n* = 2 pigs) and *via* pour-on (Ivomec Pour-On, Merial Ltd; *n* = 2 pigs) and periodic blood draws (Day 1, 3, 5, 8, 15, 22 and 28) were performed post-treatment to monitor blood levels. No mosquito live feeds were performed on pigs treated with ivermectin *via* oral and pour-on methods. Instead, required lethal doses to mosquitoes were calculated and described in the following statistical analysis.

### Statistical analysis

Key pharmacokinetic (PK) parameters were determined using non-compartmental PK analysis using STATA/MP Version 15.1 for plasma, red blood cells (RBC) and skin samples for each of the ivermectin and moxidectin-treated pigs. Details of non-compartmental PK analysis methodology in plasma, RBC and skin are described in Additional file 1: Text S1.

Key PK parameters, AUC_o-t_, AUC_o-inf_, and C_max_ were used to determine the required equivalent dose of ivermectin administered to pigs using oral and pour-on delivery in comparison to subcutaneous injection, shown to have lethal effects to mosquitoes. Microsoft Excel was used to calculate relative ivermectin bioavailability (or the proportion reaching the blood) and dose required *via* oral and pour-on delivery to achieve similar effect on vector mosquitoes *via* subcutaneous injection. Details of drug bioavailability and dose required calculation methodology are described in Additional file 1: Text S2.

Mosquito mortality resulting from MFAs was analysed using non-linear regression curve fit analysis in Graph Pad Prism v 7.00 (GraphPad Software, La Jolla California USA, www.graphpad.com) to calculate the LC_50_ of each drug. *Survminer* (https://cran.r-project.org) was used to calculate probability of survival of mosquitoes that fed on treated pigs. *Inkscape* was used to plot all figures, in R statistical software package (http://www.R-project.org).

### Mathematical modelling

Predicting the efficacy of mosquitocidal zooprophylaxis strategies in a multiple host system is contingent on understanding the distribution of blood-meals amongst hosts and what drives mosquito host choice. It is notable that, in areas of high LLIN usage in some areas of Papua New Guinea, pigs may account for up to 50% of the blood resources for malaria transmitting mosquitoes such as *An. farauti* and *An. punctulatus*. This phenomenon is attributed to net use limiting the availability of human hosts. Dogs can also be a significant alternative host and may account for up to 20% of blood meals [6, 8]. To estimate the potential impacts of ivermectin treatments, we used adapted transmission models of *P. falciparum* and *P. vivax* malaria [31] in which blood meals were taken from humans, pigs, and dogs. Both models made an assumption that ivermectin was delivered fortnightly for three months to pigs alone or to both pigs and dogs.

A discrete time (1-day time step) compartmental model was constructed to describe the key processes underlying the transmission of *P. falciparum* and *P. vivax*. The model allows for vector bites to be distributed over three host types: humans (the only infection reservoir), pigs and dogs. This is in accordance with blood-meal analyses of vectors caught from malaria endemic regions of Papua New Guinea [6, 29]. Transmission dynamics were tracked according to the following set of equations:

$$S_{t + 1}^{{}} = 1 - \left( {E_{t} + I_{t} + L_{t} } \right)$$

$$E_{t + 1}^{{}} = E_{t} + vp_{h} bZ_{t} \left( {S_{t} + L_{t} } \right) + rL_{t} - gE_{t}$$

$$I_{t + 1}^{{}} = I_{t} + gE_{t} - \left( {h + f} \right)I_{t}$$

$$L_{t + 1}^{{}} = L_{t} + hI_{t} - \left( {vp_{h} bZ_{t} + r + c} \right)L_{t}$$

$$X_{t + 1} = 1 - \left( {Y_{t} + Z_{t} } \right)$$

$$Y_{t + 1} = Y_{t} + I_{t} X_{t} p_{h} b_{v} - \left( {d + m_{1} + m_{2} + m_{3} } \right)Y_{t}$$

$$Z_{t + 1} = Z_{t} + dY_{t} - (m_{1} + m_{2} + m_{3} )Z_{t}$$

whereby, humans are compartmentalised into ‘S’usceptible, ‘E’xposed, ‘I’nfected and ‘L’atently infected; and vectors are susceptible (*X*), exposed (*Y*) or infectious (*Z*). The difference equations track the proportions among the different compartments over time (in days), t. Model parameters, their assumed values and the literature from which the values were sourced are depicted in Table 2. This set of equations describes both *P. falciparum* and *P. vivax* transmission (setting the probability of hypnozoite formation, *h*, to zero simplifies the model to an SEIS formation that is standard for *P. falciparum*).

**Table 2:** Model parameters, their values and the corresponding literature sources

	Definition (units)	Value	Source
*v*	Ratio of vectors to hosts	9	
*p*_*h*_	Proportion of bites on humans	0–1	
*b*	Transmission coefficient (vector→human) = daily bite rate (1/3) × parasite transmission probability (0.3)	0.1	[49]
*r*	Relapse rate from latent to acute infection (day^-^^1^)	0.014	[50]
*c*	Clearance rate of hypnozoite stage (day^-^^1^)	0.004	[50]
*g*	Inverse of intrinsic incubation period (day^-^^1^)	0.07	[51]
*h*	Probability of hypnozoite formation	0.63	[52]
f	Rate of clearance of active infection	0.03	[53]
*b*_*v*_	Transmission coefficient (human→vector) = daily bite rate (1/3) × parasite transmission probability (0.2)	0.07	[54, 55]
d	Extrinsic incubation period (day^-^^1^)	0.11	[56]
*m*_*1*_	Mosquito mortality rate (day^-^^1^)	0.125	[57]
*m*_*2*_	Mosquito mortality rate incurred through biting treated pig (day^-^^1^)	See text	
*m*_*3*_	Mosquito mortality rate incurred through biting treated dog (day^-^^1^)	See text	

Additional mosquito mortality incurred through endectocidal application on pigs and dogs is denoted by m_2_ and m_3_, respectively. Following previously published methods [30, 31] efficacy of the endectocide was assumed to wane over time according to: m_2_ = bite rate × proportion of bites on pigs × coverage (assumed 100%) × maximum killing efficacy (100%) × [(1 − (ln(2)/8)t]. The term in the square brackets denotes the effective period over which treated pigs are lethal to mosquitoes (i.e. ~8 days). An equivalent waning function along with the same endectocidal half-life was assumed for treated dogs (m_3_). The model does not consider sub-lethal impacts on mosquito fecundity, or the slower impacts on mosquito mortality that occur after feeding on pigs treated 15 days previously. Furthermore, this model assumes that the mosquito population is stable and density dependence at the larval stage will offset any decrease in population due to the endectocide’s reduction in fecundity. Hence, the model makes a conservative estimate of impact.

To determine the impact of mosquitocidal zooprophylaxis for different proportions of host availability, bites were randomly apportioned to humans, pigs, and dogs 500 times. This generated an average annual entomological inoculation rate (EIR) of ~100 under a *P. falciparum* scenario and ~50 under a *P. vivax* scenario.

## Additional file

**Additional file 1: Text S1.** Non-compartmental pharmacokinetic data analysis for ivermectin and moxidectin treatment in pigs through plasma, red blood cells (RBC) and skin samples. **Table S1.** Summary of non-compartmental PK data. **Figure S1.** Moxidectin concentration over time profile for the two pigs for each sample type. **Figure S2.** Natural Log of the moxidectin concentration over time profile for the two pigs for each sample type. **Figure S3.** Ivermectin concentration over time profile for the two pigs for each sample type. **Figure S4.** Natural log of the ivermectin concentration over time profile for the two pigs for each sample type. **Text S2.** The equivalent ivermectin dose determination for pour-on and oral administration to subcutaneous administration through comparisons of non-compartmental pharmacokinetic parameters. **Table S2.** Summary of Non-compartmental PK data for pigs treated with 0.6 mg/kg ivermectin. **Table S3.** Oral and pour-on ivermectin doses required for equivalence to subcutaneous injection.

## Abbreviations

MFA: membrane-feeding assay
IRS: indoor residual spraying
LLIN: long-lasting insecticide- treated net
SC: subcutaneous
EIR: entomological inoculation rate
EIP: extrinsic incubation period
C_max_: maximum concentration
AUC: area under the curve
PK: pharmacokinetic
DMSO: di-methyl sulfoxide
