# Supplementary material for: Treatment of pigs with endectocides as a complementary tool for combating malaria transmission by Anopheles farauti (s.s.) in Papua New Guinea
Source: Parasit Vectors. 2019 Mar 19;12:124. doi: 10.1186/s13071-019-3392-0 (PMC6423892; doi:10.1186/s13071-019-3392-0)
Supplement: Supplementary file 1 — Additional file 1: Text S1. Non-compartmental pharmacokinetic data analysis for ivermectin and moxidectin treatment in pigs through plasma, red blood cells (RBC) and skin samples. Table S1. Summary of non-compartmental PK data. Figure S1. Moxidectin concentration over time profile for the two pigs for each sample type. Figure S2. Natural Log of the moxidectin concentration over time profile for the two pigs for each sample type. Figure S3. Ivermectin concentration over time profile for the two pigs for each sample type. Figure S4. Natural log of the ivermectin concentration over time profile for the two pigs for each sample type. Text S2. The equivalent ivermectin dose determination for pour-on and oral administration to subcutaneous administration through comparisons of non-compartmental pharmacokinetic parameters. Table S2. Summary of Non-compartmental PK data for pigs treated with 0.6 mg/kg ivermectin. Table S3. Oral and pour-on ivermectin doses required for equivalence to subcutaneous injection. [file 13071_2019_3392_MOESM1_ESM.docx]

**Additional file 1**

**Text S1:** Non-compartmental pharmacokinetic data analysis for ivermectin and moxidectin treatment in pigs through plasma, red blood cells (RBC) and skin samples.

**Summary:** Key pharmacokinetic (PK) parameters were determined using non-compartmental PK analysis for plasma, red blood cells (RBC) and skin samples for each of the four pigs, two treated with ivermectin and another two treated with moxidectin.

**Data Description:** Data was provided for both moxidectin and ivermectin treated pigs for three different samples types: Plasma, RBC and skin. The concentration of each drug (ng/ml) was provided over a period of 29 days post treatment for ivermectin and over a period of 50 days post treatment for moxidectin. Data from the negative control pig was also provided but not required for analysis. The pre-dose concentration (time = 0 hrs) for each sample type was maintained at a value of 0 (as this was before the observed maximum concentration as per SOP guidelines). Ivermectin samples (plasma, RBC and skin) for day 29 were treated as missing where the concentration was provided as 0, in accordance with SOP guidelines for post-maximum concentrations below the limit of quantification.

**Methodology:** STATA/MP Version 15.1 was used for non-compartmental PK analysis. The PK parameters were derived using the ‘pkexamine’ command using the program default method of cubic splines for area under the curve (AUC) estimations. The key PK parameters were AUC from time 0 to last time point (AUC_0-t_), the AUC from time zero to infinity ($\mathrm{AU}C_{0-\infty}$), maximum concentration (Cmax), the time of maximum concentration (Tmax) and the elimination half-life of the drug, $t_{\frac{1}{2}}$. The PK parameters were estimated per sample type for each drug, with each pig analyzed separately.

After initial investigation of the log-concentration over time profiles, the terminal half-life was estimated using the last five concentration values. As per the SOP, the adjusted coefficient of determination (Adjusted R^2^) of the natural log concentration over time profiles used to generate the terminal half-life was used to assess the appropriateness of the number of data points used for the estimation. The terminal half-life was determined appropriate if the number of data points used for the calculation had an adjusted R-squared greater than 85%, had at least 3 data points and was calculated over a time interval of at least $2\times t_{\frac{1}{2}}$.

For all samples for both drugs the adjusted R-squared of the regression line fit for the last 5 observations in the log-concentration over time profile exceeded 85%. Sensitivity analyses were performed to assess the robustness of the results to the choice of the number of observations used to estimate the terminal phase. For both ivermectin and moxidectin treated pigs, using the last five observations corresponded with the optimal log-linear decay for both pigs for half of the sample types. Remaining samples had optimum R-squared valued using three or four points, however the adjusted R-squared using five points was still at a minimum value of 95.8%, suggesting adequacy of the terminal half-life estimate using five observations.

**Results:** The individual PK analysis results for each of the drugs and sample types are shown in Table S1, separately for each pig. Figure S1 and S2 contain the concentration over time profile and the natural log-concentration over time profile for moxidectin, respectively. Figures S3 and S4 contain the concentration over time profile and the natural log-concentration over time profile for ivermectin, respectively.

| **Table S1. Summary of Non-compartmental PK Data** | | | | | | | |
| --- | --- | --- | --- | --- | --- | --- | --- |
| **Drug** |  | **Plasma** | | **RBC** | | **Skin** | |
|  | | Pig 1 | Pig 2 | Pig 1 | Pig 2 | Pig 1 | Pig 2 |
| **Ivermectin** | 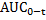   \|  \| \| --- \| | 218.3 | 275.6 | 96.1 | 117.8 | 9.0 | 14.3 |
|  | 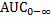 | 236.1 | 277.0 | 102.0 | 118.6 | 9.2 | 14.4 |
|  | C_max_ (ng/ml) | 26.4 | 41.7 | 10.3 | 15.6 | 0.9 | 1.9 |
|  | T_max_ (days) | 1 | 3 | 3 | 3 | 1 | 3 |
|  | t ½ (days) | 5.37 | 2.6 | 5.1 | 3.0 | 4.3 | 2.9 |
|  |  |  |  |  |  |  |  |
|  | | | | | | | |
| **Moxidectin** | 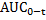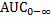 | 623.9 | 749.4 | 223.3 | 262.8 | 152.0 | 138.9 |
|  |  | 745.0 | 827.0 | 236.4 | 283.1 | 178.6 | 161.5 |
|  | C_max_ (ng/ml) | 87.5 | 79.9 | 26.1 | 20.6 | 5.2 | 6.8 |
|  | T_max_ (days) | 0.5 | 0.5 | 0.5 | 0.5 | 8 | 8 |
|  | t _½_ (days) | 21.5* | 17.4* | 11.3 | 14.1* | 18.0* | 17.6* |
|  |  |  |  |  |  |  |  |

* Note: The moxidectin terminal half-life in plasma, RBC (Pig 1 RBC excluded) and skin samples may not be appropriate as the time interval the drug was measured over was not sufficient (< 2 x t _½_ ). Therefore, caution is required when interpreting the terminal half-life for moxidectin.

**Figure S1:** Moxidectin concentration over time profile for the two pigs for each sample type

**Figure S2:** Natural Log of the moxidectin concentration over time profile for the two pigs for each sample type.

**Figure S3**: Ivermectin concentration over time profile for the two pigs for each sample type.

**Figure S4:** Natural log of the ivermectin concentration over time profile for the two pigs for each sample type.

**Text S2:** The equivalent ivermectin dose determination for pour-on and oral administration to subcutaneous administration through comparisons of non-compartmental pharmacokinetic parameters.

**Summary:** Key pharmacokinetic (PK) parameters, AUC_o-t_  , AUC_o-inf_ and C_max_ were used to determine the equivalent dose of ivermectin administered to pigs using oral and pour on methods in comparison to subcutaneous methods, shown to provide a lethal effect to mosquitos.

PK parameters were previously determined using non-compartmental PK analysis for plasma in pigs treated with 0.6mg/kg ivermectin using different delivery methods. Two pigs were treated with oral and two with pour on methods, and in a separate trial two pigs were treated with ivermectin through subcutaneous administration. For subcutaneous administration, 0.6mg/kg proved to be a sufficient dose in pigs to be lethal to mosquitos upon biting.

A summary of the PK parameters for each delivery method is provided in Table S2. Further analysis was conducted to determine the bioavailability of oral and pour-on administration, relative to subcutaneous injection, which was subsequently used to determine the equivalent dose required for oral and pour on methods.

| **Table S2. Summary of Non-compartmental PK data for pigs treated with 0.6mg/kg ivermectin** | | | |
| --- | --- | --- | --- |
| **Delivery Method** | **Oral** | **Pour On** | **Subcutaneous** |
|  | **(n=2)** | **(n=2)** | **(n=2)** |
| AUC_o-t_ | 48.3  (47.7,52.8) | 4.4  (3.4, 5.4) | 246.9  (218.3, 275.6) |
| AUC_o-inf_ | 48.5  (43.9, 53.0) | 5.9  (5.9,5.9) | 256.6  (236.1, 277.0) |
| C_max_ (ng/ml) | 19.9  (19.1,20.7) | 0.9  (0.5, 1.2) | 34.1  (26.4, 41.7) |
| T_max_ (days) | 1  (1,1) | 3  (3,3) | 2  (1,3) |
| t _½_ (days) | 1.7  (1.8, 1.7) | 6.1  (8.7,3.4) | 4  (5.4, 2.6) |
| *Data is presented as a “Mean (Pig 1, Pig 2)” | | | |

**Methodology:** Microsoft Excel was used for the analysis. The bioavailability, denoting the proportion of ivermectin reaching the blood using pour-on and oral delivery methods, was determined for each method by comparing PK measures to those of subcutaneous injection through the following formula [1]:

$$F=\frac{{AUC}_{A}}{{AUC}_{B}}\times\frac{D{ose}_{B}}{{Dose}_{A}}.$$

The bioavailability (F) for both oral and pour on were calculated using the mean of AUC_o-t_ , AUC_o-inf,_ and C_max_, provided in Table S2, and using a dose of 600µg/kg which was given to all pigs. The oral and pour on dose required to provide an equivalent PK profile to 600µg/kg ivermectin via the subcutaneous route was then determined using the relative bioavailability as follows:

$${Dose}_{A}=\frac{600\mu g/kg}{F}.$$

**Results:** The results for each of oral and pour-on delivery methods are presented in Table S3, separately for each PK measure. For oral and pour-on ivermectin delivery, 1.0-3.2mg/kg and 24.0-33.7mg/kg respectively would be required to produce an equivalent PK profile to 0.6mg/kg for subcutaneous delivery.

High dose variability can be noted between the three PK parameters used for both delivery methods. As data was available for two pigs for each of the oral, pour-on and subcutaneous delivery methods, a larger sample size would be needed to reduce the variation in the estimate of the required ivermectin dose. It should also be noted that these are theoretically the doses required, whether these are biologically plausible and whether there are other limiting factors should be taken into consideration.

| **Table S3. Oral and pour-on ivermectin doses required for equivalence to subcutaneous injection** | | | | |
| --- | --- | --- | --- | --- |
| **Delivery Method** | **PK parameter** | **Bioavailability** | **Dose Required** | |
|  |  |  | **µg/kg** | **mg/kg** |
| **Oral** | AUC_o-inf_ | 18.9% | 3177.6 | 3.2 |
|  | AUC_o-t_ | 19.5% | 3070.7 | 3.1 |
|  | C_max_ (ng/ml) | 58.4% | 1026.6 | 1.0 |
| **Pour-On** | AUC_o-inf_ | 2.3% | 26093.9 | 26.1 |
|  | AUC_o-t_ | 1.8% | 33711.3 | 33.7 |
|  | C_max_ (ng/ml) | 2.5% | 24035.3 | 24.0 |

**References:**

1. Gabrielsson, Johan; Weiner, Dan. (2006). Pharmacokinetic and pharmacodynamics data analysis: concepts and applications. Fourth Edition. Stockholm: Apotekarsocieteten. IBSN 978-9197651004
